# Vaccines in the Pharmacies?

**DOI:** 10.3390/healthcare9030269

**Published:** 2021-03-03

**Authors:** Roberto Verna

**Affiliations:** In Unam Sapientiam, Systems Biology Group, University of Rome, Viale Regina Elena, 324, 00161 Roma, Italy; roberto.verna@fondazione.uniroma1.it

In a previous article [1], I have highlighted the problem of swabs in pharmacies, stating that they have very different structures—some of which are well equipped, while others are not. Now a new problem arises with vaccines.

The request to enable pharmacies to administer COVID-19 vaccines is being raised not only in Italy, by several bipartisan political parties, but also in countries overseas [2,3]. As reported by Kate Gibson in CBS news “The White House is doubling the amount of doses being delivered to retail pharmacies across the U.S., broadening the shipments from the 1 million doses sent to 6500 pharmacies last week. The Biden administration will ship 2 million doses a week to outlets going forward and eventually will include 40,000 stores nationwide. This program will expand access in neighborhoods across the country so that people can call and make an appointment and get their shot conveniently and quickly, White House press secretary Jen Psaki said Tuesday. This is a critical, critical part of our plan” [4].

The reason for this is that pharmacies are widely distributed throughout the country and could support the NHS in its large-scale vaccination of the entire population. In itself, the reasoning is not wrong, but in this case the problem is amplified by both the differences between the available vaccines and the important differences between pharmacies. However, let us approach this step by step.

The administration of a vaccine is a medical act. As rare as they may be, the possibility of adverse reactions in the case of the inoculation of substances into the body exists and one must be prepared to face them. This cannot be done by a pharmacist. We must, in addition, consider the organization of the individual pharmacies: as reported above, some have large spaces available, while others are as small as a little apartment. This is of relevant importance in this discussion because, assuming that the pharmacy is equipped with a doctor for the administration, the space and all the devices necessary for the management of an emergency must also be warranted [5]. Of course, it would be possible to use pharmacies for vaccinations, but it would be necessary to have an organization through which the customers of the pharmacy and therefore the premises dedicated to dispensing drugs are kept perfectly separate from the premises dedicated to vaccination and from those who must be vaccinated, which should have an independent entrance and premises suitable for registration, medical history, vaccination and post-vaccine monitoring. How many pharmacies in the world are able of accomplishing this level of organization? This makes us understand how difficult it is to generalize “we can administer vaccines in pharmacies”. It is known that, for the purpose of mass vaccination, structures similar to field hospitals have been activated, in which vaccines are administered to thousands of people. In these facilities, however, vaccines are administered by doctors assisted by nurses and there are facilities for any emergencies and ambulances. This is what I was referring by “there are important differences between pharmacies”.

Of course, even pharmacies could equip themselves with ambulances in which doctors and nurses could administer the vaccines, as was already achieved for swabs; however, the aims here are not the same, even though ambulances are self-sufficient and could be placed in front of any bar or gym.

In addition, how could a pharmacy manage vaccines that need to be stored at −80 °C, which must then be brought to +4 °C and diluted before being administered? Managing very low temperatures requires more than just a refrigerator. The situation could be improved by using vaccines that need to be stored at +4 °C but the underlying issue would not change. This problem does not only affect pharmacies but also family doctors; both cannot manage storage at −80 °C, which could only be performed by hospitals or adequate facilities.

Furthermore, a vaccine administered in a pharmacy, even by a doctor, would not guarantee the protection of health, in that the person to be vaccinated could be a total stranger, of whom nothing is known. The recent discussion as to whether those who have had COVID-19 should be vaccinated is an example; only the family doctor, who knows their patients, and is really an extension of the NHS spread throughout the territory, could provide the necessary guarantees.

A final note is necessary: in Italy, pharmacies are private companies which, by agreement with the NHS, fulfil the role of dispensing drugs. Since they are private, they are therefore only subject to the moods of the owner on duty, who does not need a degree in pharmacy. 

To own a pharmacy, it is not necessary to be a pharmacist, but you must have a Pharmacist Director, who is responsible for managing the pharmacy. The professional qualification of the Pharmacist Director does not include management skills but only a degree in Pharmacy and requires professional registration (which anyone can do). An owner, however, manages his own employees that he hires by himself and that have no relationship with the NHS. Defining the contract under which pharmacists work in a pharmacy as employees as inadequate for their professionalism would be an understatement. They work hard and earn little; however, they subject to the decisions of an owner and not guaranteed by a contract with the NHS. Once authorized, the pharmacy has the right to dispense prescription drugs and over-the-counter drugs, for which a prescription is not necessary (there are numerous para-pharmacies that only dispense over-the-counter drugs).

To work as a family doctor, instead, you must first have a degree in medicine and surgery, qualification for the profession and must be personally authorized by the NHS through a personal code that must be reported on the prescriptions.

Obviously, patients do not know these subtle differences and look for the possibility of vaccination wherever it could be performed, but this could be dangerous.

Thus, my advice is: do not rush towards vaccination in pharmacies.

## Data Availability

Data are available in Riforma Giolitti (1913) art.378, TULS and art.31, RD 1706/38. Some years later, Mariotti (221/68 e 475/68) changed the terms.
